# Treatment of stress fracture of the olecranon in throwing athletes with internal fixation through a small incision

**DOI:** 10.1186/1758-2555-4-49

**Published:** 2012-12-14

**Authors:** Hiroyuki Fujioka, Kenjiro Tsunemi, Yohei Takagi, Juichi Tanaka

**Affiliations:** 1 Department of Physical Therapy, Hyogo University of Health Sciences School of Rehabilitation, 1-3-6 Minatojima, Chuo-ku, Kobe, 650-8530, Japan; 2 Department of Orthopaedic Surgery, Hyogo College of Medicine, 1-1 Mukogawa-cho, Nishinomiya, 663-8501, Japan

**Keywords:** Stress fracture, Elbow, Olecranon, Throwing athlete

## Abstract

The present study is a report of retrospective case series of stress fracture of the olecranon. Six patients presented posterior elbow pain in throwing in baseball and softball, but fracture was not diagnosed in radiographs. We detected stress fracture of the olecranon using computed tomographic (CT) scan and treated the patient with internal fixation with a headless cannulated double threaded screw through a small skin incision. All patients returned to competitive level without elbow complaints after the operation.

When throwing athletes present with unusual posterior elbow pain and no significant findings on radiographs, a CT scan examination should be performed. We recommend surgical treatment of internal fixation with a screw through a small skin incision, as a good option for stress fracture of the olecranon in order to allow early return to sports activity in competitive athletes.

## Background

Overuse injuries of the elbow joint in throwing athletes, such as osteochondritis dissecans of the capitelum humeri at the lateral side of the elbow joint and insufficiency of the medial collateral ligament at the medial side of the elbow joint, are common in sports medicine. Osteoshondrosis of the capitelum humeri are caused by impaction of the humeroradial joint and injuries of the medial collateral ligament are caused by excessive traction force due to valgus stress applied to the elbow during throwing [1,2].

By contrast, injuries to the posterior side of the elbow joint, which are less common, are known as stress fracture and epiphiseal injury of the olecranon and osetophyte formation of the olecranon. These injuries are caused by impingement of the olecraonon in the olecranon fossa of the humerus during valgus extension overload stress in pitching and by excessive pulling of the triceps brachii muscle on the olecranon during the acceleration phase of the throwing [3-6]. There are three types of fractures of the olecranon: the first type is a fracture of the tip of the olecranon osteophyte due to impingement of the olecranon fossa [3,6]; the second type is a fracture of the growth plate preventing the closure of the olecranon epiphysis seen in skeletally immature patients [4,5,7]; the third type is a straight or oblique fracture line in the middle third of the olecranon seen in the skeletally mature patients and this type of stress fracture of the olecranon caused by repetitive stress forces have been infrequently reported as a cause of elbow pain in adult throwing athletes [8-10].

In this report, we present the clinical features of a stress fracture of the olecranon in six skeletally mature patients who were treated with internal fixation through a small incision.

## Case presentation

Six adolescent throwing athletes, 5 baseball pitchers and 1 softball pitcher, were included in this retrospective study (Table 1). All patients felt posterior elbow pain on throwing without any major causes. Although they suspended throwing for several weeks, the symptoms did not decrease and they visited us. Tenderness was detected at the posterior site of the olecranon, however, range of motion of the elbow was normal. In all cases, radiographs of the elbow showed neither osteophyte formation nor osteosclerotic change, but computed tomographic (CT) scan revealed a fracture line of the olecranon.

**Table 1:** Summary of the cases

**Case**	**Age**	**Sex**	**Sport Played**	**Duration From Onset to Surgery**	**Duration until Return to Competition Level after Surgery**
1	18	M	Baseball Pitcher	1 year and 4 months	4 months
2	16	M	Baseball Pitcher	5 months	6 months
3	17	M	Baseball Pitcher	7 months	6 months
4	18	F	Softball Pitcher	6 months	4 months
5	19	M	Baseball Pitcher	6 months	6 months
6	21	M	Baseball Pitcher	2 weeks	6 months

All patients were treated with internal fixation using DTJ large screw through a small incision and the implant was not removed.

Although all patients reduced their sports activities for several weeks before visited us, elbow pain continued. Therefore we treated the patients surgically with internal fixation through small skin incision. The average duration from the onset to surgery was 6.8 months (range: 2 weeks to 1 year and 4 months). The average duration of follow-up was 2 years and 4 months (range: 2 to 3 years).

All patients were surgically treated with internal fixation with DTJ (Double Threaded screw Japan, Meira, Japan) large screw, which is a cannulated double threaded headless screw. Under general anesthesia, on the lateral decubitus position, a longitudinal 2 or 3 cm skin incision was made on the proximal site of the olecranon. A guide wire was fluoroscopically inserted to the ulna, from the lateral tip of the olecranon to the medial distal cortex of the ulna, crossing the fracture site. After drilling and tapping, an appropriate size screw was inserted through a small skin incision with neither exposure of the fracture site nor bone graft at the fracture site. The distal thread reached the medial cortex of the ulna and the proximal thread was located under the proximal end of the olecranon.

After two weeks immobilization with long arm cast, range of motion and muscle strengthening exercises, such as elbow extension and flexion exercise, forearm pronation and supination exercises, and wrist extension and flexion exercise, were started. Once full range of motion was obtained (4–6 weeks postoperatively) a gradual return to throwing program was started. At postoperative 4 months, the patients were allowed to return to competition level.

All patients had neither elbow pain during throwing nor loss of range of motion of the elbow and could return to competitive level, with almost the same performance level as before injury, at 6 months after surgery. The implant did not need to be removed in all patient.

### Representative case (case 2)

**Figure 1:**
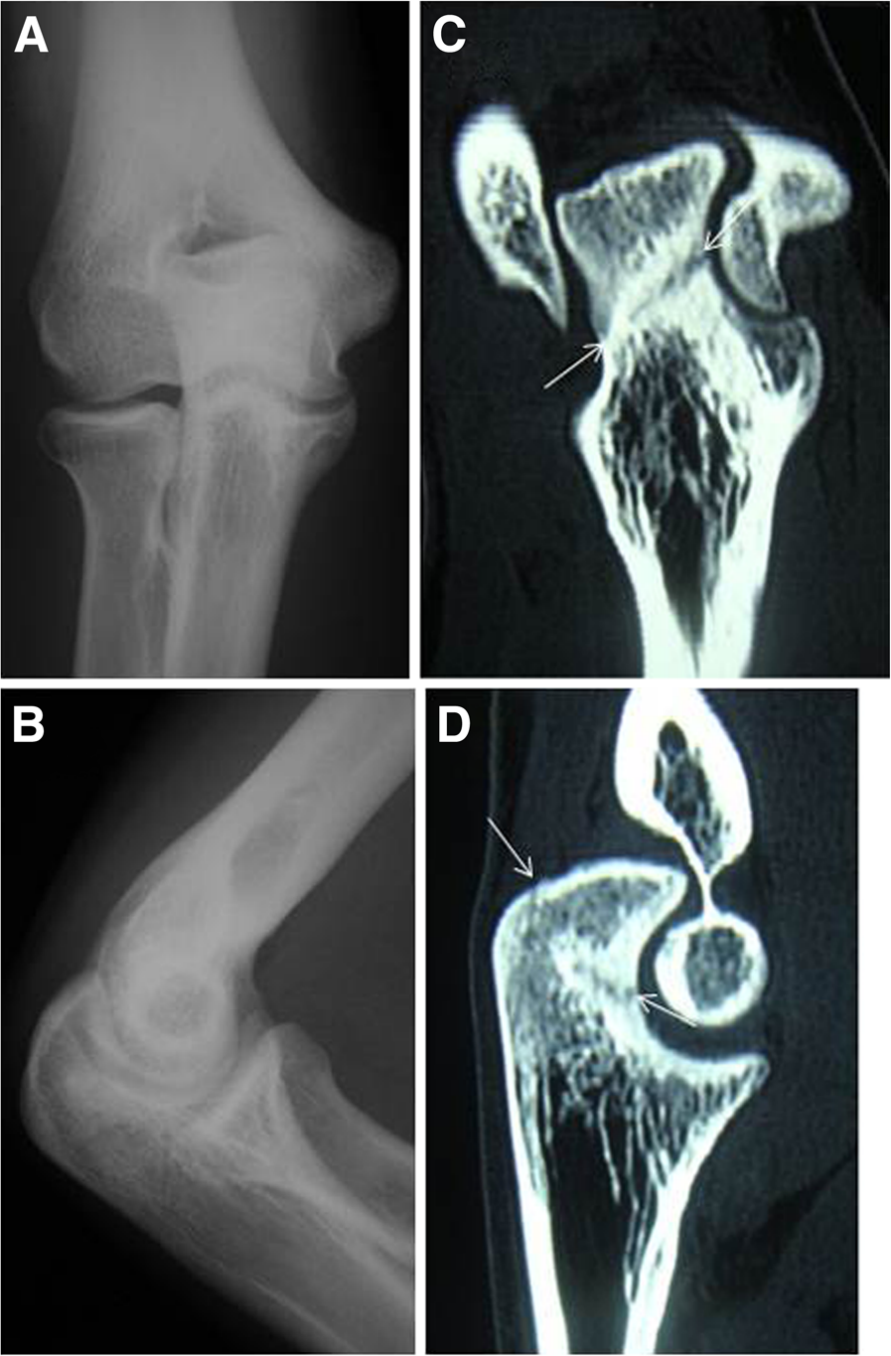
**Case 2.** Radiographs and CT scan of the elbow before surgery. There were no significant signs such as fracture or osteoarthritic change on anteroposterior (**A**) and lateral (**B**) views of radiographs. On the frontal (**C**) and sagittal plane (**D**) of CT scan, non-displaced and straight oblique fracture line (arrow), from medial proximal to lateral distal at the middle third of the olecranon, was detected.

**Figure 2:**
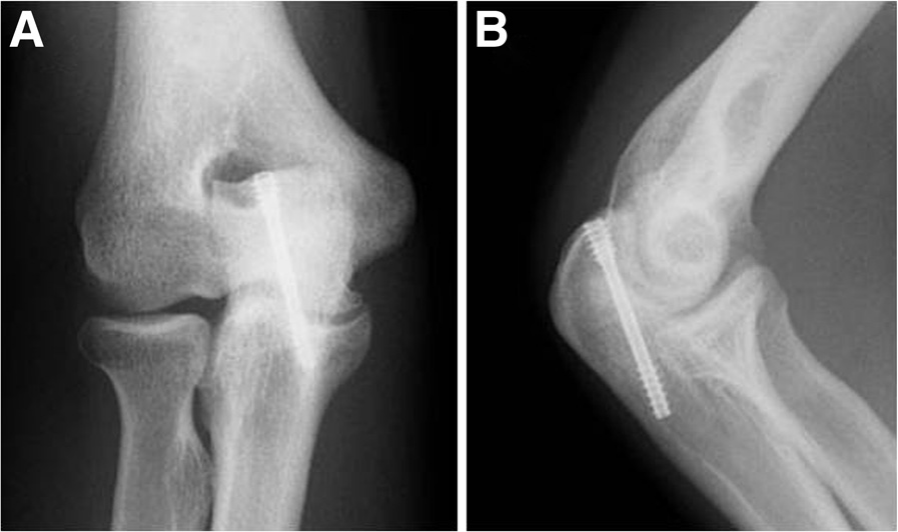
**Case 2.** Radiographs after surgery. The screw was inserted through a small incision with neither fracture site open nor bone graft and the fracture site was fixed with the screw on anteroposterior (**A**) and lateral (**B**) views of the radiographs. The distal thread of the screw reached the medial cortex of the ulna and the proximal thread was located in the cancellous bone of the olecranon.

The patient, a 16 year-old-man, was a high school baseball pitcher. He felt pain in the posterior aspects of the right elbow during throwing. Pain increased progressively until he was unable to throw. Physical examination revealed tenderness on the olecranon of the involved right elbow, however, there was no limitation of range of motion in the elbow. There were no significant signs such as fracture or osteoarthritic change on anteroposterior and lateral views of the radiographs (Figure 1A,B). However, CT scan clearly revealed a non-displaced and straight oblique fracture line from the medial to the lateral at the middle third of the olecranon (Figure 1C,D). We diagnosed the patient with stress fracture of the olecranon and treated him with internal fixation using DTJ large screw as described above (Figure 2). The operated elbow was immobilized with long arm cast for two weeks after surgery. Thereafter, the patient started rehabilitation program of active range of motion and strengthening muscles. The patient returned to competitive baseball without elbow symptoms 6 months after the internal fixation. 

## Discussion

Stress fractures are partial or complete fractures of a bone resulting from an inability to withstand stress applied in a repeated manner [11]. Stress fractures of the lower extremities are common injuries in physically active athletes, however, stress fractures of the upper extremities are not common. In the upper extremities, stress fracture of the first rib, the olecranon, and the metacarpal bone have been reported as sports related injuries [11]. Because stress fracture is caused by overuse and fatigue of the surrounding musculature, most stress fractures heal with conservative treatment, such as rest and structured rehabilitation. In order to prevent throwing-related elbow pain in adolescent overhead athletes, improving rotator cuff strength with posterior shoulder muscle strengthening and scapular stabilization are critical parts of elbow rehabilitation [12,13].

Nuber and Diment presented two cases of baseball players with non-displaced stress fractures detected as straight oblique fracture line at the middle third of the olecranon that were treated conservatively [10]. However they suggested that the long period of complete rest and immobilization for treatment was needed and that the fractures had the potential to displace due to traction force by the triceps brachii muscle.

Hulkko et al. reported that two javelin throwers at international level had oblique stress fractures at the middle third of the olecranon and were surgically treated with a tension band and two Kirschner wires [8]. These fractures healed in 4 months with no symptoms, however, one patient suffered a re-fracture and was treated surgically again. They recommend stress fractures of the olecranon be treated surgically in javelin throwers because of the high risk of delayed union. Nakaji et al. reported an olecranon stress fracture treated with open reduction and internal fixation with tension band wiring, however, the patient was suffering from re-fracture of the olecranon after implant removal and a revision surgery with internal fixation using a screw was performed [9]. They recommend surgical treatment of internal fixation with screw in competitive baseball players.

In order to detect stress fracture or occult fracture, CT scan, magnetic resonance imaging (MRI), and bone scintigraphy are all useful methods [14,15]. In athletes with early tibial stress injuries, multidetector CT scan has a good performance in the identification of cortical abnormality such as osteopenia, which is the earliest sign of fatigue damage of the cortical bone, and MRI has a good performance to evaluate soft tissue damage, perioseteal edema, and bone marrow edema [14]. In occult scaphoid fracture, multidetector CT scan is superior to dipect cortical involvement [15]. In our report, patients presented with pain at the posterior site of the elbow joint on throwing and fractures of the olecranon could not be detected on the radiographs but were visible on CT scans. Because stress fracture presents with gradual onset of pain in the elbow on throwing over a period of several weeks and the fracture line cannot usually be seen on radiographs, appropriate diagnosis may be delayed. CT scan is a useful examination to diagnose this problem.

In this report, all patients were treated with internal fixation with headless screw and the screws were not necessary to be removed in any patients. All patients had neither elbow pain during throwing nor loss of range of motion of the elbow and could return to competitive level.

## Conclusions

When throwing athletes present with unusual posterior elbow pain and no significant findings on radiographs, a CT scan should be taken. In the competitive athletes required high performance that the fracture of the olecranon is detected on a CT scan, the fracture should be surgically treated with internal fixation with a screw through a small incision. Internal fixation using a headless double threaded cannulated screw was useful for dependable and early return to competitive level.

### Consent

Written informed consent was obtained from all patients for publication of this case report and any accompanying images.

## Abbreviations

CT: Computed tomography; MRI: Magnetic resonance imaging.

## Competing interests

The authors delicate that they have no competing interests.

## Authors’ contributions

All authors contributed towards the treatment of the patients and in the manuscript preparation and approved the final manuscript.
